# ‘My adopted daughter and then my second wife died of kuru’

**DOI:** 10.1098/rstb.2008.4003

**Published:** 2008-11-27

**Authors:** Atenamu Bavasa

**Affiliations:** Ketabi Village, c/o Papua New Guinea Institute of Medical ResearchPO Box 60, Goroka, EHP 441, Papua New Guinea

I was in my thirties and had a son by the name of Anua when Dr Carleton Gajdusek came into the Purosa Valley to do research on kuru. Carleton picked Anua from among the boys to work with him. When Carleton, Anua and others went on patrol, I assisted them by carrying field equipment and rations for two to three weeks. I also assisted by explaining to the village people why we were there in their village, how we wanted to examine the sick kuru patients and what help we tried to give them.

I was asked by Carleton to stay back in the village to take care of his house and equipment. After 3 years, my adopted daughter and then my second wife died of kuru, so I gave up my job to take care of the children left behind.

I am pleased that the Kuru Project is continuing the kuru research work with the people of Okapa. The two main groups, the Atigina and the Pamusagina, have worked hard and assisted the medical scientific officers in their studies from the beginning and they continue to assist the kuru research workers today. Now I am working with Michael Alpers and Jerome Whitfield to research the stories of our ancestors and the cultural practices of the Pamusa people.

